# Type 1 diabetes mellitus abrogates compensatory augmentation of myocardial neuregulin-1β/ErbB in response to myocardial infarction resulting in worsening heart failure

**DOI:** 10.1186/1475-2840-12-52

**Published:** 2013-03-27

**Authors:** Oghenerukevwe Odiete, Ewa A Konik, Douglas B Sawyer, Michael F Hill

**Affiliations:** 1 Department of Medicine, Division of Cardiovascular Medicine, Vanderbilt University Medical Center, Nashville, TN 37232, USA

**Keywords:** ErbB2, ErbB4, Heart failure, MDM2, Myocardial infarction, Neuregulin-1, Type 1 diabetes mellitus

## Abstract

**Background:**

Diabetes mellitus (DM) patients surviving myocardial infarction (MI) exhibit a substantially higher incidence of subsequent heart failure (HF). Neuregulin (NRG)-1 and erythroblastic leukemia viral oncogene homolog (ErbB) receptors have been shown to play a critical role in maintenance of cardiac function. However, whether myocardial NRG-1/ErbB is altered during post-MI HF associated with DM remains unknown. The aim of this study was to determine the impact of type 1 DM on the myocardial NRG-1/ErbB system following MI in relation to residual left ventricular (LV) function.

**Methods:**

Type 1 DM was induced in rats via administration of streptozotocin (65 mg/kg, i.p.). Control rats were injected with citrate buffer (vehicle) only. Two weeks after induction of type 1 DM, MI was produced in DM and non-DM rats by ligation of the left coronary artery. Sham MI rats underwent the same surgical procedure with the exception that the left coronary artery was not ligated. At 4 weeks after surgery, residual *in vivo* LV function was assessed via echocardiography. Myocardial protein expression of NRG-1β, ErbB2 and ErbB4 receptors, and MDM2 (a downstream signaling pathway induced by NRG-1 that has been implicated in cell survival) was assessed in the remaining, viable LV myocardium by Western blotting. Changes in ErbB receptor localization in the surviving LV myocardium of diabetic and non-diabetic post-MI rats was determined using immunohistochemistry techniques.

**Results:**

At 4 weeks post-MI, echocardiography revealed that LV fractional shortening (FS) and LV ejection fraction (EF) were significantly lower in the DM + MI group compared to the MI group (LVFS: 17.9 ± 0.7 vs. 25.2 ± 2.2; LVEF: 35.5 ± 1.4 vs. 47.5 ± 3.5, respectively; *P* < 0.05), indicating an increased functional severity of HF among the DM + MI rats. Up-regulation of NRG-1β and ErbB2 protein expression in the MI group was abrogated in the DM + MI group concurrent with degradation of MDM2, a downstream negative regulator of p53. ErbB2 and ErbB4 receptors re-localized to cardiac myocyte nuclei in failing type 1 diabetic post-MI hearts.

**Conclusions:**

Type 1 DM prevents compensatory up-regulation of myocardial NRG-1/ErbB after MI coincident with an increased severity of HF.

## Background

Diabetes mellitus (DM) patients surviving myocardial infarction (MI) have substantially higher cardiovascular morbidity and mortality than those without DM [1]. The poor clinical prognosis of these patients has been linked primarily to the more frequent development of heart failure (HF) [2,3]. Despite the higher risk for DM patients to develop HF after MI, our understanding of the mechanism(s) by which DM exacerbates the incidence of this syndrome remains infantile.

Neuregulin (NRG)-1, along with the erythroblastic leukemia viral oncogene homolog (ErbB) 2, 3, and 4 receptor tyrosine kinases through which NRG-1 ligands signal, have been shown to play an essential role in cardiac development [4,5] and maintenance of the adult heart [6]. In the adult heart, at least 3 different NRG-1α isoforms and 8 NRG-1β isoforms are expressed, with the β isoform being 10 to 100 times more bioactive. Endothelial cells of the endocardium release NRG-1 and it has been shown to promote the growth and survival of cardiomyocytes in culture through the activation of ErbB2 and ErbB4 receptors [7,8]. Cardiac-specific deletion of ErbB2 and ErbB4 in mice has been reported to result in the development of dilated cardiomyopathy [6,9]. *In vivo*, endothelium-selective deletion of NRG-1 has been demonstrated to worsen post-ischemic contractile recovery after coronary artery ligation [10].

Recently, changes in the NRG-1/ErbB system have been documented to occur in the setting of type 1 DM. In rats with type 1 diabetic cardiomyopathy, mRNA expression and phosphorylation of the ErbB2 and ErbB4 receptors as well as NRG-1 protein expression were observed to be markedly decreased [11]. Administration of NRG-1 to type 1 diabetic cardiomyopathy rats resulted in a significant improvement in cardiac function [12]. These findings suggest that alterations in NRG/ErbB signaling may play a pathophysiological role in the development of cardiac dysfunction associated with type 1 DM. However, studies examining the impact of DM per se on the myocardial NRG-1/ErbB system in response to MI remain absent.

Accordingly, the aim of the present study was to determine whether DM adversely affects myocardial NRG-1/ErbB after MI and thereby enhances the development of HF. Using a rat model of HF due to MI by coronary ligation in the presence of STZ-induced DM, a type 1 model, we assessed the protein expression profiles of NRG-1β, ErbB2 and ErbB4 receptors, MDM2 (a downstream signaling pathway induced by NRG-1 that has been implicated in cell survival), as well as changes in ErbB receptor localization in the surviving LV myocardium of diabetic post-MI rats in relation to residual LV function. We chose to utilize a type 1 DM model even though the vast majority of acute myocardial infarct patients are type 2 diabetics for several reasons. First, the risk of mortality from ischemic heart disease is exceptionally high in patients with type 1 DM [13]. Second, considering that patients with type 1 DM are younger at the onset of their disease, they stand to lose many more life-years from cardiovascular disease, which includes MI and HF, than do those with type 2 DM [14]. Lastly, published work on DM and the heart has been related mainly to type 2 DM despite the fact that type 1 DM imparts substantial risk for cardiac disease [15]. As such, we specifically employed a type 1 DM model in the present study to directly address the disparity in studies on cardiovascular disease in type 1 DM.

## Methods

### Experimental animals

Sprague–Dawley rats were obtained from Harlan (Indianapolis, IN). Experiments were performed according to a protocol approved by the Institutional Animal Care and Use Committee at Vanderbilt University Medical Center (Protocol ID#: M/09/322). The investigation conforms to the *Guide for the Care and Use of Laboratory Animals* published by the US National Institutes of Health.

### Induction of Type 1 DM

Type 1 DM was induced in male Sprague–Dawley rats (200–224 g body weight) by administering a single intraperitoneal injection of STZ (65 mg/kg body wt) prepared daily in citrate buffer pH 4.5 for maximal stability. The control vehicle (CV) group was injected with an equal volume of the vehicle. Development of DM was confirmed 48 hours later by the presence of glycosuria (>2000 mg/dl) along with polyuria as described previously [16]. Two weeks after induction of DM, diabetic and non-diabetic rats underwent surgical induction of MI.

### Induction of MI

Rats were anaesthetized intraperitoneally with Nembutal (40 mg/kg). Rats were then rapidly intubated and mechanically ventilated by a constant volume small animal ventilator (Model 683, Harvard Apparatus). A left thoracotomy was performed at the fourth intercostal space and the LAD was ligated at the level immediately below the bottom of the left atrium by irreversible tightening of a 6–0 suture loop. The bottom of the left atrium was used as a demarcation point to ensure consistent placement of the ligature and resultant reproducibility of similar infarct sizes among the groups of animals. This demarcation point was also used to avoid ligation of the LAD too proximally to its origin which would lead to fatal cardiac arrhythmias. MI was confirmed by regional cyanosis of the myocardial surface distal to the suture, accompanied by S-T segment elevation on the electrocardiogram (ECG). Sham MI (SMI) animals underwent the same surgical procedure with the exception that the LAD was not ligated. Rats were allowed to recover and then used at 4 weeks post-MI for different studies.

### Assessment of *In vivo* residual LV function by echocardiography

Transthoracic echocardiographic images of hearts from all groups of rats were obtained at 4 weeks post-MI using an ultra high-resolution ultrasound scanner (Vevo 2100; VisualSonics) under nembutal anesthesia. For M-mode recordings, the parasternal short-axis view was used to image the heart in two dimensions at the level of the papillary muscles. LV fractional shortening (FS) and ejection fraction (EF) were recorded along with LV cavity dimensions (end-diastolic and end-systolic).

### Tissue harvest

Following echocardiographic assessment, hearts from all groups of rats were rapidly excised and perfused with ice-cold physiological saline and weighed. The atria and ventricles were dissected and the infarcted (scar) and non-infarcted regions of the LV was separated, weighed, and frozen in liquid nitrogen. The non-infarcted LV tissue was used for all molecular analyses.

Pieces of tissues from the lungs and liver were removed and weighed. For the determination of dry weight, these were placed in an oven at 65°C until a constant weight was reached. Ratios of wet to dry weight were calculated for both lungs and liver.

### Western blot analysis

LV tissue was homogenized in 1X RIPA lysis buffer (Millipore), supplemented with protease inhibitor cocktail (Roche). 50 μg of protein was loaded onto a PAGE using precasted gradient gel 4-12% NuPage gels (Invitrogen). After electrophoresis, proteins were transferred to an Immun-Blot PVDF membrane using a Semi-Dry transfer apparatus. Membranes were blocked using PBST-5% BSA and then incubated overnight at 4°C with antibodies directed against ErbB2, ErbB4 and murine double minute 2 (MDM2) (clone F-11, clone C-18, and H-221, respectively) (Santa Cruz). NRG-1β and α-tubulin (Santa Cruz) were also analyzed by western blot. Secondary horseradish peroxidase-conjugated antibody (Cell Signaling) was applied for 1 h at room temperature. Blots were visualized using the SuperSignal West Pico chemiluminiscent substrate (Thermo Scientific) and analyzed with NIH Image J densitometry software.

### Immunohistochemistry

LV specimens were embedded in O.C.T. medium (Tissue-Tek), and 5–10 μM sections were prepared with a cryotome. Nonspecific binding was blocked with 0.1% Bovine serum albumin in PBS for 30 min, and coverslips were incubated overnight with anti-ErbB2 and anti-ErbB4 (Santa Cruz) and antibody against N-Cadherin (Sigma). A secondary antibody conjugated with FITC and TXRD-conjugated phalloidin (Molecular Probes) was added for 1 h. Coverslips were mounted using Vectashield (Vector laboratories). DAPI (Vector laboratories) was used according to manufacturer instructions for nuclear staining. Images were generated with LSM710 confocal microscope (CIRC, Vanderbilt University).

### Measurement of blood glucose

Prior to MI surgery and at the time of sacrifice, blood samples were collected from the tail vein for the determination of blood glucose levels using Accutrend Plus test strips and meter (Roche).

### Protein determination and statistical analysis

Protein concentrations of LV tissue samples were determined using the Modified Lowry Protein Assay Reagent Kit (Pierce). Data were expressed as the mean ± S.E.M. Group means were compared by one-way analysis of variance (ANOVA), and ANOVA followed by Bonferroni’s test was used to identify differences between multiple groups. Comparisons between two groups were performed by Student’s *t*-test. Values of *P* < 0.05 were considered statistically significant.

## Results

### Animal characteristics

Mortality following induction of MI was higher among the DM + MI group compared to the MI group (47% vs. 33%). Blood glucose levels were significantly elevated in the DM and DM + MI groups of rats compared with the non-diabetic animal groups (Table 1). Animals in the DM and DM + MI groups showed significantly less weight gain as compared to their non-diabetic counterparts (Table 1). Heart weight was increased in the MI and DM + MI groups (Table 1). No significant differences in the weight of myocardial scar caused by the infarction were observed between MI and DM + MI groups of rats (Table 1). The wet to dry weight ratios of the lungs was observed to be elevated only in the DM + MI animals (Table 1), suggesting that severe HF was present only in this group.

**Table 1:** Animal characteristics at 4 weeks post-MI

**Parameter**	**CV**	**DM**	**Sham MI**	**MI**	**DM + MI**
Body Weight, g	365 ± 10.6	225 ± 11.6^*^	356 ± 7.0	376 ± 9.4^#^	227 ± 7.7*^†‡^
Blood Glucose, mg/dl	152 ± 6.1	547 ± 20.2^*^	142 ± 7.3	163 ± 5.2^#^	549 ± 13.9^*†‡^
Total Heart Weight, g	1.2 ± 0.03	0.9 ± 0.08^*^	1.3 ± 0.03	1.5 ±0.05^†#^	1.3 ± 0.06^#‡^
Scar Weight, g	ND	ND	ND	0.20 ±0.03	0.19 ± 0.02
Lung Wet/Dry	4.8 ± 0.03	4.8 ± 0.08	5.1 ± 0.11	5.0 ± 0.09	5.3 ± 0.08^*#‡^
Liver Wet/Dry	3.3 ± 0.12	3.0 ± 0.11	3.2 ± 0.05	3.1 ± 0.12	3.3 ± 0.04

Values are mean ± standard error of the mean of four to six animals. ND: not detectable. CV, control vehicle; DM, diabetes mellitus; Sham MI, sham myocardial infarction, MI, myocardial infarction; DM + MI, diabetes mellitus + myocardial infarction. ^*^P < 0.05 vs. CV; ^†^*P* < 0.05 vs. Sham MI; ^#^*P* < 0.05 vs. DM; ^‡^*P* < 0.05 vs. MI.

### Type 1 DM exacerbates post-MI HF independent of adverse remodeling

**Figure 1:**
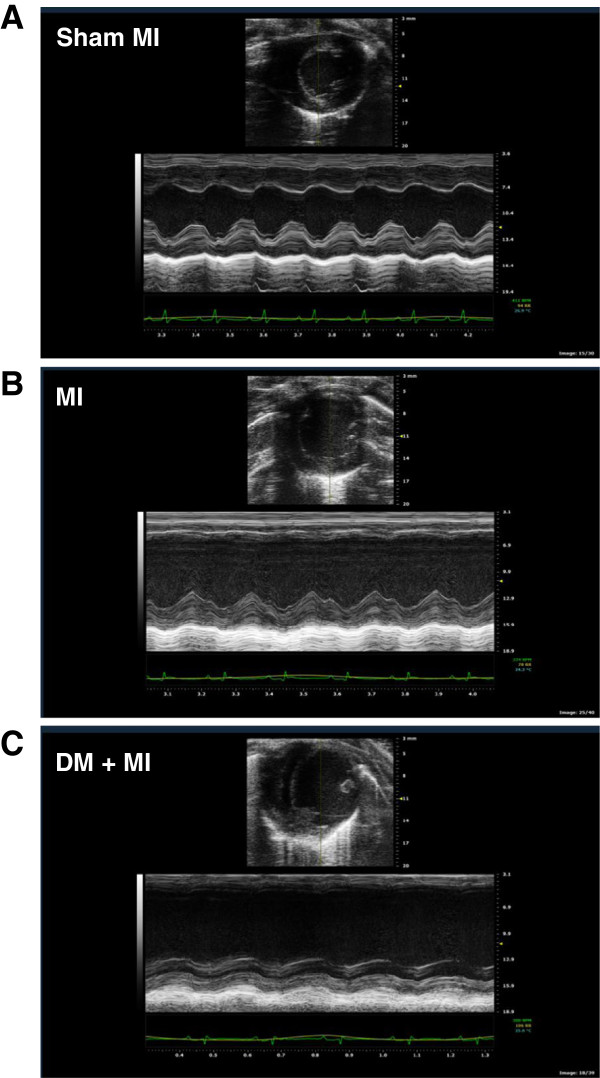
**Representative echocardiographic images of diabetic and non-diabetic rat hearts at 4 weeks after MI.** Images consist of 2-dimensional echocardiography at parasternal short-axis plane (top portion) and M-mode short axis of LV (bottom portion). (**A**): Sham MI; (**B**): MI; (**C**): DM + MI.

Echocardiographic studies revealed differences in residual LV function between MI and DM + MI groups of rats. Representative echocardiograms obtained from a sham MI, MI and DM + MI rat are shown in Figure 1. Echocardiographic data are summarized in Table 2. LVFS and LVEF were significantly lower in the DM + MI group compared to the MI group, indicating an increased functional severity of HF in the diabetic post-MI group. Although a statistically significant reduction in LVEF and LVFS was also observed in the DM group without MI, the magnitudes of such decreases were significantly less as compared to the DM + MI group. Heart rate was significantly lower in the DM and DM + MI groups (Table 2).

**Table 2:** **Echocardiographic assessment of *In Vivo *residual LV function at 4 weeks post-MI**

**Parameter**	**CV**	**DM**	**Sham MI**	**MI**	**DM + MI**
LVFS,%	43.4 ± 1.9	34.3 ± 1.1^*^	40.4 ± 1.0	25.2 ± 2.2^†#^	17.9 ± 0.7^*†#‡^
LVEF,%	73.3 ± 2.3	61.5 ± 1.5^*^	69.5 ± 1.2	47.5 ± 3.5^†#^	35.5 ± 1.4^*†#‡^
LVIDd, mm	6.3 ± 0.22	7.7 ± 0.20^*^	6.6 ± 0.24	9.3 ± 0.55^†#^	9.2 ± 0.67^*†^
LVIDs, mm	3.6 ± 0.23	5.1 ± 0.17^*^	4.2 ± 0.19	7.0 ± 0.55^†#^	7.8 ± 0.37^*†#^
Heart Rate, bpm	350 ± 23.5	241 ± 9.0^*^	376 ± 16.5	361 ± 11.1^#^	299 ± 9.3^†#‡^

LVFS, left ventricular fractional shortening; LVEF, left ventricular ejection fraction; LVIDd, left ventricular internal dimension, diastolic; LVIDs, left ventricular internal dimension, systolic. Values are mean ± standard error of the mean of four to six animals. ^*^P < 0.05 vs. CV; ^†^*P* < 0.05 vs. Sham MI; ^#^*P* < 0.05 vs. DM; ^‡^*P* < 0.05 vs. MI.

As shown in Table 2, MI and DM + MI groups of rats had a significantly larger LV chamber size as compared to the sham MI group as indicated by significant increases in LVIDd and LVIDs. However, no differences in LVIDd and LVIDs were observed between the MI and DM + MI groups themselves. This observation is consistent with the findings from the Survival and Ventricular Enlargement (SAVE) echocardiographic substudy which demonstrated that after MI, diabetes is not associated with greater ventricular enlargement [17]. Although an increase in LVIDd and LVIDs was observed in DM rats in the absence of MI, the extent of such increases were significantly less pronounced compared with the DM + MI group of rats.

### Up-regulation of NRG-1β in post-MI heart is abolished by the presence of type 1 DM

**Figure 2:**
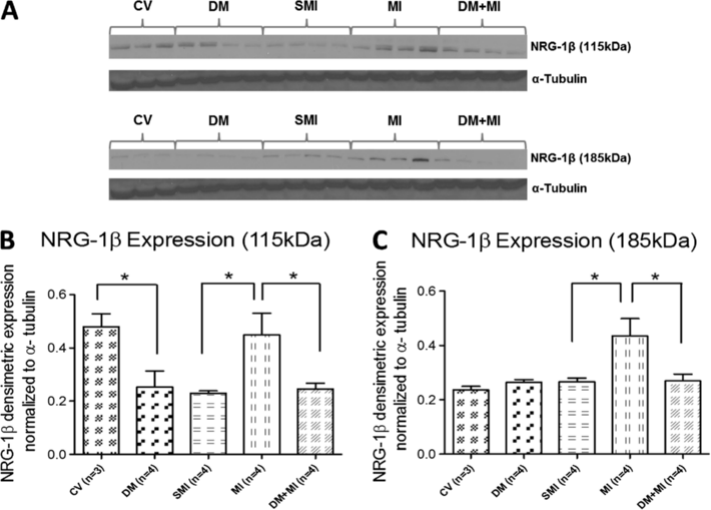
**Myocardial NRG-1β protein expression in MI and DM + MI groups of rats.** (**A**): Representative Western blots for 115 kDa and 185 kDa fragments of NRG-1β, (**B**): Densitometric analysis of the immunoreactivity of the 115 kDa fragment of NRG-1β, (**C**): Densitometric analysis of the immunoreactivity of the 185 kDa fragment of NRG-1β. In all samples, the densitometric measurements of NRG-1β immunoreactivity were normalized to α-tubulin expression. *P < .01.

Myocardial NRG-1β protein expression showed differences between the MI and DM + MI groups of rats (Figure 2). A 96% and 63% increase in the protein expression of the 115 kDa and 185 kDa fragments of NRG-1β, respectively, was observed in the MI group compared to the sham MI rats (Figuire 2B + 2C). In contrast, there was a 44% and 39% decrease in the expression of the 115 kDa and 185 kDa fragments of NRG-1β, respectively, in the DM + MI group vs. the MI group (Figure 2B + 2C). Interestingly, in the DM group without MI, a significant decrease in the expression of only the 115 kDa fragment of NRG-1β was observed compared to the CV group (Figure 2B). We also observed that the 115 kDa NRG-1β fragment was down-regulated with sham surgery.

### Up-regulation of myocardial ErbB2 after MI is suppressed by concurrent type 1 DM

**Figure 3:**
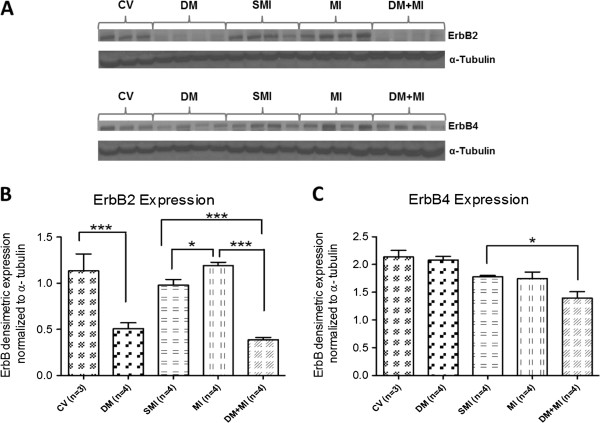
**Myocardial ErbB2 and ErbB4 receptor protein expression in MI and DM + MI rats.** (**A**): Representative Western blots for ErbB2 and ErbB4, (**B**): Densitometric analysis of ErbB2 receptor immunoreactivity, (**C**): Densitometric analysis of ErbB4 receptor immunoreactivity. In all samples, the densitometric measurements of ErbB2 and ErbB4 immunoreactivity were normalized to α-tubulin expression. *P = .02; ***P < .0001.

Myocardial ErbB2 and ErbB4 receptor protein expression was analyzed in all groups of rats (Figure 3). Compared to the sham MI group, a 21% increase in myocardial ErbB2 protein expression was observed in the MI group (Figure 3B) while no differences in protein expression was observed with respect to ErbB4 (Figure 3C). However, in the DM + MI group, a 67% decrease in the protein expression level of the ErbB2 receptor was observed compared to the MI group (Figure 3B). Although a decrease in the protein expression level of the ErbB4 receptor was also observed in the DM + MI group compared to the MI group (Figure 3C), this reduction did not reach statistical significance. The expression levels of ErbB2, but not ErbB4, were significantly decreased in DM hearts without MI when compared with the CV group (Figure 3B).

### MDM2 is down-regulated in the failing type 1 diabetic post-infarction heart in parallel with increased MDM2 degradation

**Figure 4:**
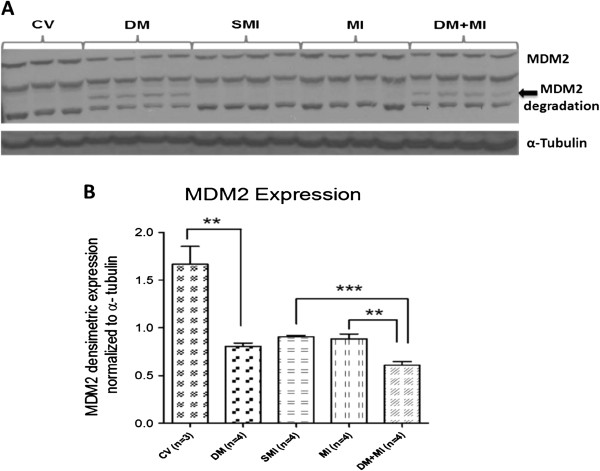
**Myocardial protein expression of MDM2 in MI and DM + MI rats.** (**A**): Representative Western blot for MDM2, (**B**): Densitometric analysis of MDM2 immunoreactivity. Arrow shows MDM2 degradation product. In all samples, the densitometric measurement of MDM2 immunoreactivity was normalized to α-tubulin expression. **P = .003; ***P < .0001.

Myocardial MDM2 protein expression was analyzed in all groups of rats (Figure 4). Cardiac expression of MDM2 was significantly decreased in DM + MI rats vs. sham MI and MI groups of rats (33% and 31% decrease, respectively) whereas such down-regulation of MDM2 was not observed in non-diabetic MI rats (Figure 4B). The reduction in MDM2 protein expression in the DM + MI group was associated with evidence of MDM2 degradation (Figure 4A). Similar results were observed in DM rats without MI. MDM2 expression levels were also observed to be lower with sham surgery.

### Localization of ErbB receptors is disrupted in the type 1 diabetic post-MI heart with advanced HF

**Figure 5:**
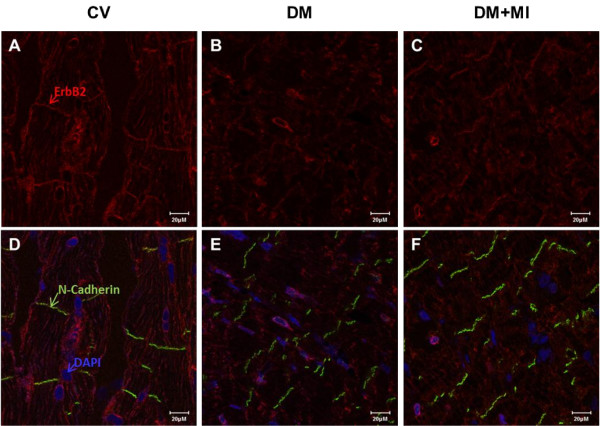
**Immunolocalization of ErbB2 receptor in failing diabetic post-MI rat heart.** Cross-sections of rat heart tissue (**A-F**) were immunostained for ErbB2 (red), N-Cadherin (green), and DAPI (blue), visualized by confocal microscopy. Controls lacking primary anti-ErbB2 antibody showed minimal background staining (data not shown). Bar represents 20 μM. Immunolocalization images representative of 5–6 animals per group.

**Figure 6:**
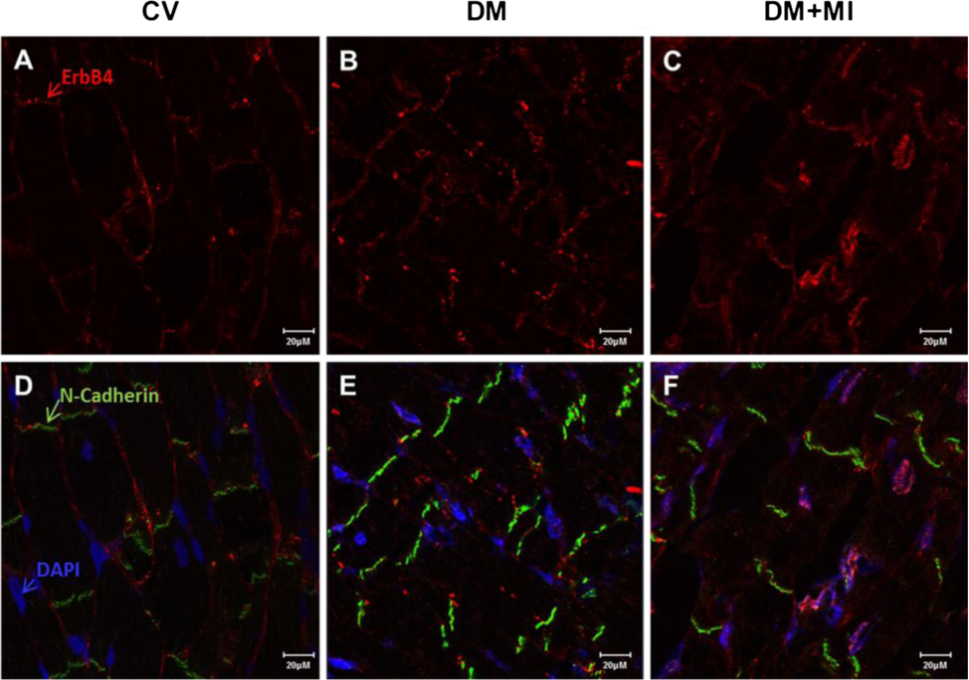
**Immunolocalization of ErbB4 receptor in failing diabetic post-MI rat heart.** Cross-sections of rat heart tissue (**A-F**) were immunostained for ErbB4 (red), N-Cadherin (green), and DAPI (blue), visualized by confocal microscopy. Controls lacking primary anti-ErbB4 antibody showed minimal background staining (data not shown). Bar represents 20 μM. Immunolocalization images representative of 5–6 animals per group.

We examined the cellular localization of ErbB2 and ErbB4 in heart tissue by immunostaining. In the control vehicle heart, ErbB2 and ErbB4 were localized primarily in the cellular membrane of myocytes including the intercalated disk (Figure 5D + Figure 6D, respectively). Cellular membrane staining for ErbB2 were less clear in the DM heart (i.e. outline of myocyte) (Figure 5E) which became more diffuse in the DM + MI heart (Figure 5F). The DM and DM + MI heart demonstrated a prominent peri-nuclear localization of ErbB2 (Figure 5E + 5F, respectively). Nuclear ErbB4 staining was apparent in some myocyte nuclei from DM rat hearts (Figure 6E). However, in the DM + MI heart, more prominent nuclear ErbB4 staining was observed (Figure 6F).

## Discussion

Little is known about perturbations in biological processes that take place in the diabetic heart following MI. The major novel findings of the present study are: (1) up-regulation of myocardial NRG-1β protein expression following MI is abrogated by the concomitant presence of type 1 DM; (2) up-regulation of myocardial ErbB2 protein expression following MI is abrogated by the concomitant presence of type 1 DM; (3) degradation and decreased protein expression of MDM2 occurs in type 1 diabetic post-MI hearts; (4) ErbB2 and ErbB4 re-localize to the nucleus from the intercalated disk in cardiac myocytes of failing type 1 diabetic post-MI hearts; and (5) these alterations in the myocardial NRG-1β/ErbB pathway in the type 1 diabetic post-infarction heart are associated with an increased functional severity of HF. These findings shed new light on the underlying mechanisms that contribute to the impaired response to infarction in diabetic hearts. Furthermore, our data support the concept that augmentation of myocardial NRG-1β/ErbB is an important cardioprotective adaptation to MI that is lost in the presence of type 1 DM.

Numerous studies have demonstrated the cardioprotective role of the NRG-1/ErbB signaling pathway. Ischemia-reperfusion injury is associated with activation of NRG-1β/ErbB4 signaling that mediates recovery [18]. NRG-1/ErbB signaling is also activated during the early stages of HF [19], similar to what we observed in the post-MI heart. Reduced NRG-1/ErbB signaling is associated with impaired myocyte resistance to the cytotoxic effects of neurohormonal stimuli such as β1-adrenergic receptor activation [20]. The demonstration in the present study that type 1 DM prevents up-regulation of myocardial NRG-1β/ErbB expression in response to MI concomitant with an increased severity of HF further illustrates that the NRG-1β/ErbB system in the heart appears to be a local cardioprotective signaling system. Moreover, failure of adaptive tuning of myocardial NRG-1β/ErbB to MI in the presence of DM may explain, in part, how the diabetic post-MI heart is rendered more prone to accelerated HF.

In the present study, we found that STZ-diabetic rats without MI exhibited reduced myocardial expression of the 115 kDa fragment of NRG-1β and ErbB2 receptor. This is consistent with a recent report showing down-regulation of ErbB receptor mRNA expression and NRG-1 protein expression in LV myocardium of STZ-diabetic rats [11]. Collectively, these observations point to DM *per se* as the adverse factor and suggest that the suppression of the NRG-1β/ErbB pathway induced by STZ-diabetes precludes harnessing this system as a compensatory mechanism to help in recovery of myocardial function after superimposition of cardiac injury involving MI. One question that arises from these findings is: How does DM down-regulate myocardial NRG-1/ErbB expression? In this regard, NRG-1 mRNA and protein expression are known to be down-regulated by angiotensin II [21]. In light of the fact that the activity of the Renin-Angiotensin-Aldosterone System (RAAS) is increased in DM, over-activation of RAAS may serve as a potential mechanism by which DM suppresses NRG-1 expression. Physiological metabolic stress of β-adrenergic receptor stimulation by norepinephrine causes ErbB2 degradation [20]. Increased catecholamine levels in cardiac tissue are a common finding in DM [22] and hence may also be a potential mechanism underlying reduced ErbB receptor expression.

Having found that ErbB2 and ErbB4 protein expression was down-regulated in type 1 diabetic hearts with advanced post-MI HF, we also looked at their localization in cardiomyocytes. Our results demonstrate that ErbB2 localizes to the peri-nuclear area and ErbB4 localizes to the nucleus in cardiac myocytes of failing type 1 diabetic post-MI hearts, thereby confirming our Western blot analysis. Previous studies have demonstrated that the increased localization of ErbB4 to the nucleus in adult rat ventricular myocytes may be an indicator of cell stress response [23], a finding that has also been observed in other receptor tyrosine kinases [24]. Whether nuclear localization of ErbB receptors affects their availability and/or ability to bind NRG-1 ligands and thereby contribute to the obliteration of compensatory NRG-1/ErbB signaling in the diabetic heart after MI is equally important from a therapeutic standpoint and warrants further investigation.

**Figure 7:**
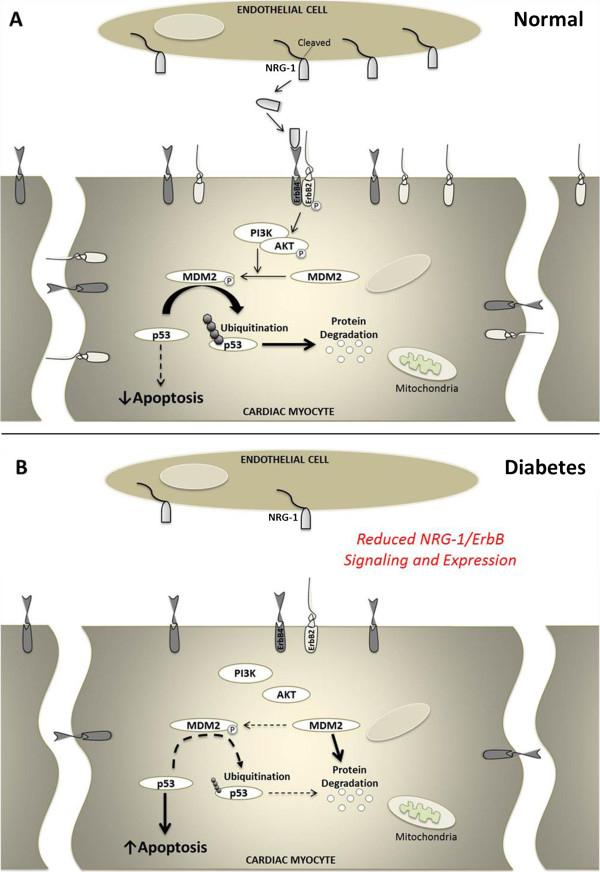
**Schematic of the NRG-1/ErbB pathway in the normal and diabetic heart.** (**A**) Schematic of the NRG-1/ErbB pathway in the normal heart. NRG-1/ErbB activates a number of downstream signaling cascades, such as PI3-kinase, AKT, and MDM2. Activation of MDM2 causes ubiquitination and resultant degradation of p53 resulting in the latency of p53 action. (**B**) Proposed molecular mechanism of action by which type 1 DM suppresses the myocardial NRG-1/ErbB pathway. Degradation and decreased expression of MDM2 induced by type 1 DM may appreciably reduce the inhibitory effects that MDM2 typically has on p53 and as such may represent a potential molecular mechanism for diabetes-induced cardiac cell death and associated cardiomyopathy.

MDM2 is activated by ErbB2 signaling and under normal circumstances is the principal negative regulator of p53 via ubiquitination and subsequent degradation [25] (Figure 7A). Since we found that ErbB2 protein expression was down-regulated in type 1 diabetic hearts with advanced post-MI HF, we examined whether MDM2 was also suppressed. Our data shows for the first time that the deleterious effects of type 1 DM on the myocardial NRG-1/ErbB pathway extend downstream of the ErbB2 receptor as evidenced by degradation and decreased expression of MDM2 in type 1 diabetic hearts with and without MI. This result may be of particularly important significance in that it may help to further our understanding of how DM induces myocardial apoptotic cell death, a key initiating factor for the development of diabetic cardiomyopathy. Although myocardial apoptosis was not measured in cardiac tissue sections in the present study, in cardiac myocytes, hyperglycemia has been documented to activate p53 leading to myocyte cell death [26]. *In vivo*, cardiac expressions of p53 have been observed to be markedly increased in STZ-diabetic animals [27,28] resultantly promoting enhanced myocyte apoptosis [28]. Together, these observations raise the interesting possibility that the enhanced cardiac apoptotic cell death imposed by DM may stem from its actions on the MDM2-p53 pathway whereby DM represses the inhibitory effects that MDM2 typically has on p53. This proposed molecular mechanism of action by DM is illustrated in Figure 7B. Further studies, such as MDM2 over-expression, will be needed to confirm whether DM may additionally execute its deleterious effects on the myocardium via alteration of the MDM2/p53 axis.

Derangements in fatty acid metabolism inherent to DM may be responsible for and linked to diabetes-induced changes in myocardial NRG-1/ErbB signaling. Fatty acid accumulation increases cardiac p53 expression in association with cardiac dysfunction in DM [27]. Marked up-regulation of several enzymes involved in fatty acid metabolism occurs in the hearts of STZ-diabetic rats [16]. *In vitro*, it is interesting that treatment of neonatal rat cardiac myocytes with the saturated fatty acid palmitate disrupts NRG-1β activation of the PI3-kinase/Akt pathway leading to an increase in p53 expression concurrent with degradation of MDM2 [29]. The phosphorylation of Akt has been reported to be markedly decreased in the hearts of diabetic animals [30,31]. Taken together, these observations raise the intriguing possibility that diabetes-induced increases in the levels of circulating fatty acids may contribute to alterations in myocardial NRG-1β/ErbB signaling and biology.

The significantly lower HR observed in the STZ-diabetic groups of rats is consistent with our previous observations [16,32] as well as prior reports in the literature [33-37] and may be due to cardiac autonomic neuropathy [33,34]. Although reductions in heart rate are routinely associated with cardiac depression, studies undertaking functional comparison at comparable heart rates via pacing in STZ-diabetic rats have reported similar alterations in myocardial contractility despite correcting for heart rate [38,39]. Therefore, the LV systolic dysfunction observed in the DM and DM + MI groups cannot merely be the result of the much reduced heart rate and instead points to greater impairment of myocardial contractile function.

The tendency of clinical trials to focus on cardiovascular disease in type 2 DM (as opposed to type 1 DM) in spite of the fact that the age-adjusted relative risk for cardiovascular disease in type 1 DM far exceeds that of type 2 DM has resulted in an under-appreciated link between type 1 DM and cardiac disease [15]. As such, in the present study, we aimed to bridge this gap by employing un-treated STZ-induced diabetes, a well-established model of type 1 DM that closely recapitulates uncontrolled hyperglycemia due to absolute insulin deficiency. While possible confounding effects of STZ administration have been suggested to occur acutely, the half-life of the drug is ~15 minutes, and it is cleared from the organs within 4 hours [40]. Given that the data reported in the present study was obtained at 6 weeks after STZ injection, the results reported herein are likely related to the development of diabetes. Furthermore, the reversal of accompanying mechanical changes in cardiac function induced by STZ with insulin [41,42] renders the possibility that the abnormalities observed among the diabetic animals in the present study being due to drug toxicity, rather than the diabetic condition itself, extremely unlikely.

### Study limitations

First, our data do not provide direct proof of a cause-and-effect relationship between the impairment of myocardial NRG-1β/ErbB up-regulation after MI and the exacerbation of LV failure by type 1 DM. However, in a recent study, administration of recombinant human NRG-1 (rhNRG-1) to STZ-induced diabetic cardiomyopathy rats rescued the depressed cardiac function [12] suggesting that derangements in NRG-1/ErbB may play a causal role in the decreased cardiac function brought about by DM. Further studies examining whether these same effects of NRG therapy can be recapitulated in diabetic hearts with co-existing ischemic injury are required to answer this crucial question that was not examined in the present study. Second, the results of the present study do not delineate how MDM2 is being degraded. While beyond the scope of this study, future experiments are needed to fully elucidate the detailed mechanisms by which DM induces degradation of MDM2 in the heart. Lastly, the 115 kDa NRG-1β fragment and MDM2 were observed to be down-regulated with sham surgery. NRG and MDM2 expression levels may be negatively influenced by the inflammatory microenvironment [43,44] which could explain this result. Nonetheless, since the sham surgery group served as a control for our MI and DM + MI groups, the differences in NRG-1β and MDM2 protein expression found in each of the MI and DM + MI groups cannot be attributed simply to inflammatory responses invoked by the thoracotomy itself.

## Conclusions

Our results suggest that loss of compensatory up-regulation of myocardial NRG-1β/ErbB coupled with disruption of ErbB receptor localization may be responsible, at least in part, for the greater incidence and severity of HF in post-MI patients with co-existing DM. In addition, our finding of degradation and decreased expression of MDM2 in failing type 1 diabetic infarcted hearts implicates this downstream ErbB2 receptor-associated pathway as part of the molecular pathophysiology of aggravated HF in the type 1 diabetic post-infarction heart. Taken together, these observations provide innovative and valuable mechanistic insights into the diabetes-related deficit in response to MI. Approaches aimed at restoring the myocardial NRG-1/ErbB pathway in the diabetic post-MI heart may prove to be beneficial in preventing and/or ameliorating the subsequent development of HF.

## Abbreviations

DM: Diabetes mellitus; ECG: Electrocardiogram; ErbB: Erythroblastic leukemia viral oncogene homolog; HF: Heart failure; HR: Heart rate; LV: Left ventricular; LVFS: Left ventricular fractional shortening; LVEF: Left ventricular ejection fraction; LVIDd: Left ventricular internal dimension, diastolic; LVIDs: Left ventricular internal dimension, systolic; MDM2: Murine double minute 2; MI: Myocardial infarction; NRG-1: Neuregulin-1; SEM: Standard error of mean; STZ: Streptozotocin.

## Competing interests

The authors declare that they have no competing interests.

## Authors’ contributions

OO contributed to the conception and design of the study, acquisition and analysis of data, and drafted the article. EAK contributed to acquisition and analysis of immunohistochemistry data and revising the article critically for important intellectual content. DBS contributed to analysis and interpretation of data and revising the article critically for important intellectual content. MFH contributed to the conception and design of the study, analysis and interpretation of data, and revising the article critically for important intellectual content. All authors read and approved the final manuscript.
